# Courtesy stigma among primary caregivers of children with autism spectrum disorder in eastern China

**DOI:** 10.3389/fpsyt.2023.1236025

**Published:** 2023-11-17

**Authors:** Xu Chen, Jiao Tong, Bochen Jiang, Shan Ma, Xin Wang, Xun Sun, Yuan Liu, Dongmei Yan, Leilei Wang

**Affiliations:** ^1^Lianyungang Maternal and Child Health Hospital, Lianyungang, China; ^2^Shandong University, Jinan, China

**Keywords:** courtesy stigma, primary caregivers, children, ASD, China

## Abstract

**Introduction:**

The experience and perception of stigma is a common problem among primary caregivers of children with autism spectrum disorder (ASD), and has a profound adverse impact on primary caregivers and children with ASD; however, few studies have explored courtesy stigma among primary caregivers of children with ASD in the Chinese context. The aim of this study was to explore the status of courtesy stigma among primary caregivers of children with ASD in Lianyungang, Jiangsu Province, Eastern China, and to conduct in-depth analysis of its predictors from multiple perspectives.

**Methods:**

An institution-based multi-center cross-sectional survey was conducted in the rehabilitation department of a large specialized hospital and 10 rehabilitation centers for children with special needs in Lianyungang, Jiangsu Province, Eastern China, from October 2022 to February 2023. A structured questionnaire to assess child-related factors, primary caregiver-related factors, courtesy stigma, general self-efficacy, and social support, was used to collect data. Predictors of courtesy stigma among primary caregivers of children with ASD were identified by linear regression.

**Results:**

A total of 428 primary caregivers of children with ASD were recruited. The mean ± standard deviation (SD) score for courtesy stigma was 7.49 ± 4.13. Multiple linear regression analysis revealed that primary caregivers of children with ASD who were not too satisfied with their current marital status (*β* = 1.21, 95% CI: 0.34–2.08, *p* < 0.05) were more likely to have a high courtesy stigma; however, significantly lower courtesy stigma was observed in primary caregivers of children with ASD who were not picky eaters (*β* = −1.33, 95% CI: −2.08 – –0.58, *p* < 0.05), and who reported low level challenge in caring for children with ASD (*β* = −1.16, 95% CI: −2.20 – –0.12, *p* < 0.05), good general self-efficacy (*β* = −0.16, 95% CI: −0.25 – –0.06, *p* < 0.05), and good social support (*β* = −0.04, 95% CI: −0.08 – –0.01, *p* < 0.05).

**Conclusion:**

There is a high level of courtesy stigma among primary caregivers of children with ASD in eastern China, and it is affected by numerous factors. More resources should be directed to groups that are more likely to experience stigma.

## Introduction

Autism spectrum disorder (ASD) is a neurodevelopmental condition, characterized by social and communication impairments and limited repetitive behavioral features, the prevalence of which is increasing worldwide (1, 2). A recent meta-analysis reported that the prevalence of ASD in China is 26 per 10,000 people and rising (3). Due to insufficient comprehension and misunderstanding of ASD in mainstream society, the characteristics of children with ASD often lead to discrimination toward them (4). Notably, some characteristics of children with ASD are often also blamed on their caregivers, who may be accused of being unskilled and inefficient in caring for their children; thus, caregivers are also exposed to stigma (5–7). Primary caregivers of children with ASD often experience stigma, including courtesy stigma and affiliate stigma, particularly in school and community settings, and more so than primary caregivers of children with intellectual and physical disabilities (6, 8). Courtesy or affiliate stigma are types of social stigma experienced due to association with a stigmatized person (9). In courtesy stigma, primary caregivers experience stigma due to sharing interpersonal relationships with an affected child, while affiliate stigma focuses on negative stereotypes internalized by the primary caregiver (4). Although there are high levels of stigma among primary caregivers of children with ASD, there has been insufficient research into stigma, particularly courtesy stigma, in this context.

Stigma can lead to low self-esteem and social withdrawal of primary caregivers of children with ASD (10). Further, it can lead to reduced health-seeking behavior and even impaired caregiving, which can affect the mood and behavior of children (11, 12). Longitudinal studies have confirmed that parental experiences of discrimination have a significant impact on internalized and externalized features in children with ASD (13). Primary caregivers of children with ASD who experience stigma also have increased levels of psychological distress, including sadness, helplessness, hopelessness, tension, worry, irritability, and fear, which can all have negative impacts on their relationships with their spouses and children (13, 14); however, empirical research on stigma interventions among primary caregivers of children with ASD is inadequate (4). Therefore, study of this phenomenon and factors predictive of stigma among primary caregivers of children with ASD is necessary to, to provide a reference for formulation of intervention measures.

In recent years, there has been an increased research focus on stigma in primary caregivers of children with ASD. Studies conducted in multiple countries have shown that child-related factors, including the sex, age, and severity of symptoms of children, are predictors of stigma among primary caregivers of children with ASD (15–18). In terms of primary caregiver-related factors, resilience, relationship with children, depression, anxiety, self-compassion, cognitive fusion, burden, self-esteem, trait mindfulness, and perceived stress, marital satisfaction of the primary caregiver were correlated with stigma (17–24). In addition, unmet ASD care needs, family structure, and the number of children with ASD in the family were also factors influencing stigma (25). Marital satisfaction was found to be associated with higher negative emotions and stigma in mothers of children with ASD (26, 27). Despite these findings, research to date has been confined to investigation of factors influencing stigma from limited perspectives. Notably, studies from other populations have found that caregivers of picky eaters face criticism from others and experience stigma because their parenting skills are often considered ineffective (28). Income, self-efficacy, and social support are also important predictors of stigma (29–33). Income can affect the parenting ability of children with ASD. Caregivers with higher income may have better knowledge of behavior management through education and access to ASD services (34), and thus better manage their children and reduce the negative views of others. Evidence already suggests that low income is a predictor of emotional problems in mothers of children with ASD (35). Self-efficacy refers to a relatively stable, confident personality trait and a sense of optimism about one’s ability to face life’s difficulties (36). Caregivers with higher self-efficacy are more likely to deal with difficulties with confidence and may experience lower levels of stigma. Family support as part of social support has been elucidated to be significantly negatively associated with stigma (37). However, there is a lack of research on the relationship between social support and courtesy stigma. Perceived social support may be key to improving caregiver resilience during the diagnosis and management of children with ASD (38). Lack of social support may feel more social exclusion and isolation, increasing stigma. Further, family caregivers with high caregiving burden experienced more severe stigma (39). In addition, challenging parenting experiences predict anxiety, depression, and stress symptoms in mothers of children with ASD (40). Caregivers with challenging parenting experiences may encounter more difficulties in the process of caregiving, such as managing children’s behavioral problems, others’ perceptions of children, and parenting confidence. However, whether challenging parenting experiences influence stigma is unclear. Based on these publications, we inferred that the reported variables may impact courtesy stigma among primary caregivers of children with ASD.

It is difficult for primary caregivers of children with ASD to avoid struggling with stigma. Identifying predictors of stigma among primary caregivers of children with ASD could help to identify populations at risk and develop effective interventions; however, stigma may also vary depending on socio-cultural contexts, such as political systems, etiquette customs, and value systems (41). Most research on stigma to date has been limited to western developed countries. Numerous researchers have noted that further research on stigma among primary caregivers of children with ASD in developing countries is needed to understand the experience of the primary caregivers of children with ASD in different contexts (15, 42). Stigma may be more prevalent in Asian societies, due to the concept of “face” and stigma socialization (4). “Face” describes the “self-image” that a person experiences because of the evaluation of a particular situation by others. People may have the feeling of losing their “face,” due to negative social evaluation (43). In addition, stigma may be more prominent in China’s collectivist culture (41), as individuals who deviate from societal norms in collectivist cultures tend to experience more stigmatization (44). Different cultural practices in China may also contribute to heightened levels of stigma relative to those in other countries (37). Due to the emphasis on social acceptance and validation in Chinese culture, individuals tend to exhibit heightened sensitivity towards negative public perceptions; however, there has been insufficient research on courtesy stigma among primary caregivers of children with ASD in eastern China.

### Aims of the current study

The aims of the current study were to explore the present status of courtesy stigma among primary caregivers of children with ASD in Lianyungang, Jiangsu Province, Eastern China, and to conduct deep analysis of the effects of child-related factors (sex, age, duration of rehabilitation, comorbidities, severity of symptoms, single child, and picky eater), primary caregiver-related factors (sex, age, education level, relationship to children with ASD, area of residence, employment status, monthly family income, satisfaction with marital status, levels of challenge in caring for children, and economic burden), general self-efficacy, and social support on courtesy stigma. Our findings will increase the current limited knowledge of courtesy stigma among primary caregivers of children with ASD in China, and will be instructive for the design and development of effective interventions to reduce courtesy stigma among primary caregivers of children with ASD.

## Materials and methods

### Study design

An institution-based multi-center cross-sectional survey was conducted from October 2022 to February 2023 in the rehabilitation department of a large specialized hospital and 10 rehabilitation centers for children with special needs in Lianyungang, Jiangsu Province, Eastern China; these facilities are responsible for providing services for children with ASD.

### Participants

At the study sites, primary caregivers (including fathers, mothers, and grandparents) who reported that they lived with, and provided the most care services for, a child with ASD were invited to participate in the study. The inclusion criteria were as follows: (1) primary caregivers of children diagnosed with ASD by an occupational clinician, according to the diagnostic criteria of the American Psychiatric Association’s Diagnostic and Statistical Manual of Mental Disorders, Fifth Edition (DSM-5), which are widely used in Chinese hospitals; (2) children with ASD were ≤ 12 years old; (3) the primary caregiver was ≥18 years old; (4) able to understand the research process and the content of the questionnaire; (5) consent to participate in the study. In order to exclude stigma caused by other serious physical illness and neurological diseases, to obtain the stigma of primary caregivers caused by children with ASD, and to consider the quality of questionnaire completion, we set exclusion criteria. The exclusion criteria were: (1) children with severe physical illness or other neurological diseases; (2) the primary caregiver had a history of any psychiatric disorder diagnosed by an occupational clinician. The minimum sample size required was calculated using the single-population proportion formula. Due to the lack of relevant previous studies at the study site, to obtain the maximum sample size, we used the prevalence of stigma as 50% (*p* = 50%), 95% confidence interval (CI), margin error of 5%, and non-response rate of 10%. The required sample size was calculated as 423. A systematic random sampling technique was employed to select participants. To obtain more reliable conclusions, a total of 450 primary caregivers were invited to participate in the survey, and 450 questionnaires were collected, with a response rate of 100%. Among them, 22 questionnaires were excluded as invalid, because they were not fully completed due to time constraints. A total of 428 valid questionnaires were included in the study (effective response rate, 95.1%). The characteristics of the sample are presented in Table 1.

**Table 1:** Characteristics of sample (*N* = 428).

Variables	*n*	%
Sex of children		
Male	298	69.63
Female	130	30.37
Age of children (years)		
<6	286	66.82
≥6	142	33.18
Duration of rehabilitation		
<6 months	140	32.71
6–12 months	64	14.95
>1 years	224	52.34
Comorbidities		
Yes	28	6.54
No	400	93.46
Severity of symptoms		
Mild	129	30.14
Moderate	218	50.93
Severe	81	18.93
Single child		
Yes	202	47.20
No	226	52.80
Picky eater		
Yes	256	59.81
No	172	40.19
Sex of primary caregiver		
Male	154	35.98
Female	274	64.02
Age of primary caregiver (years)		
≤30	127	29.67
31–45	268	62.62
>45	33	7.71
Education		
Below college degree	252	58.88
College or above degree	176	41.12
Relationship to child with ASD		
Father	148	34.58
Mother	261	60.98
Grandparents	19	4.44
Area of residence		
Urban	233	54.44
Rural	195	45.56
Employment status		
Employed	215	50.23
Unemployed	213	49.77
Monthly family income (yuan)		
≤5,000	182	42.52
5,001–10,000	170	39.72
>10,000	76	17.76
Satisfaction with marital status		
Satisfaction	323	75.47
Not too satisfaction	105	24.53
Levels of challenge in caring for children		
High	366	85.51
Low	62	14.49
Economic burden		
Severe	283	66.12
Mild	145	33.88

### Procedure

One primary caregiver for each child was asked to complete a questionnaire. The investigators were five people with a master’s degree or above from a large specialized hospital who had received standardized training. The investigators informed the primary caregivers of children with ASD about the purpose of the current study, the process, and the benefits of participation, etc. They also provided explanations to the primary caregivers when questions in the questionnaire required clarification. In appreciation for participation, we presented a small gift to each primary caregiver who completed the questionnaire.

### Measures

A structured questionnaire, designed based on an extensive review of the relevant literature and consultation with experts in the field, was used for data collection. In addition, we conducted a pre-survey at the study site. According to the findings of the pre-survey and additional expert evaluation, the questionnaire was revised and improved. The questionnaire included five parts: child-related factors, primary caregiver-related factors, courtesy stigma, general self-efficacy, and social support. Both child-related factors and primary caregiver-related factors were measured using a single item. Child-related factors included sex, age, duration of rehabilitation, comorbidities, severity of symptoms, single child, and picky eater (referring to the rejection of numerous of familiar or unfamiliar foods and intake of an inadequate variety of foods). The severity of symptoms was classified as mild, moderate, or severe and was completed by the primary caregivers based on a clinician diagnosis. Picky eater was assessed by asking “Does your child refuse to eat certain foods or eat very little (e.g., vegetables, fruits)?.” Primary caregiver-related factors included sex, age, education level, relationship to children with ASD, area of residence, employment status, monthly family income, satisfaction with marital status, levels of challenge in caring for children, and economic burden. Satisfaction with marital status was measured by asking “Are you satisfied with your current marital status?.” Levels of challenge in caring for children were assessed by asking “How challenging do you think it is to take care of your child?.” Economic burden was determined by asking “How do you feel about the economic burden of your child’s treatment on you?”

Courtesy stigma was measured using the Perceived Courtesy Stigma Scale (PCSS), modified from the Devaluation of Consumer Families Scale (DCFS) (45). The scale contains 7 items, and each item is scored on a 4-point Likert scale, ranging from “strongly disagree” to “strongly agree,” with scores of 0 to 3, respectively. The total score is the sum of the scores for each item, with higher scores indicating greater courtesy stigma. The effectiveness of the PCSS has been demonstrated among primary caregivers of children with ASD (41). In the current study, the Cronbach’s α coefficient value for the PCSS was 0.895.

General self-efficacy was assessed using the General Self-Efficacy Scale (GSES). Self-efficacy refers to an individual’s perception or belief about whether they can take appropriate actions in the environment (46). The GSES consists of 10 items, and each item is scored on a 4-point Likert scale ranging from “not at all true” to “exactly true” with scores of 1 to 4, respectively. The scores of each item were summed to obtain the total score, which ranged from 10 to 40. A higher total score indicates greater general self-efficacy. The scale has been shown to have good reliability and validity (47), and its Cronbach’s α coefficient value in the current study was 0.911.

Social support was assessed using the Multidimensional Scale of Perceived Social Support (MSPSS), which comprises three dimensions: family support, friend support, and other support (48). The scale consists of 12 items, with four items included in each dimension. Each item is scored on a 7-point Likert scale ranging from “very strongly disagree” to “very strongly agree” with scores of 1 to 7, respectively. The total score is the sum of the scores for each item, with higher scores indicating greater levels of social support. The scale has been widely used in a variety of populations in China and other countries, including primary caregivers of children with ASD, and has good reliability and validity (34, 49). In the current study, the Cronbach’s α coefficient value was 0.956.

### Ethical approval

This study was approved by the Ethics Committee of Lianyungang Maternal and Child Health Hospital. This study was conducted in line with the principles of the Declaration of Helsinki. All participants were informed of the purpose of the study, the confidentiality of the data, and the right to withdraw from the study at any time before participating in the study. All participants provided written informed consent.

### Data analysis

Eligible questionnaires with no missing items were coded and entered into a database created using EpiData 3.1 software (EpiData Association, Odense, Denmark). All data were exported to SPSS21.0 software (IBM Corporation, Armonk, State of New York) for statistical analysis. Continuous data were tested for normality by calculating kurtosis and skewness coefficients (50), and were found to be normally distributed. Continuous data are described as mean and standard deviation (SD), and categorical data are described as frequency and percentage. The significance of differences in courtesy stigma scores among primary caregivers of children with ASD were evaluated using *t*-tests and one-way ANOVA. Pearson correlation analysis was used to assess correlations between general self-efficacy, social support, and courtesy stigma among primary caregivers of children with ASD. Variables with statistical significance (*p* < 0.05) in univariate analysis were included in a multiple linear regression model, to exclude the influence of confounding factors and identify independent factors influencing courtesy stigma among primary caregivers of children with ASD. All comparisons were two-sided and *p* < 0.05 was considered to indicate statistical significance.

## Results

### Current status of courtesy stigma among primary caregivers of children with ASD

Among the 428 primary caregivers of children with ASD, the mean ± SD courtesy stigma score was 7.49 ± 4.13, and the mean ± SD score for each item was 1.07 ± 0.59.

### Child-related factors

Of the participants’ children with ASD, a large proportion were male (69.63%) and < 6 years old (66.82%). More than half of children (52.34%) had undergone rehabilitation treatment for >1 year, and a small proportion (6.54%) had co-morbid diseases. Almost one-fifth of the children (18.93%) had severe symptoms, almost half (47.20%) were only children, and approximately three-fifths (59.81%) were picky eaters (Table 1). Univariate analyses showed that primary caregiver courtesy stigma scores were significantly associated with severity of symptoms and picky eaters (*p* < 0.05) (Table 2).

**Table 2:** Relationship between child-related factors and courtesy stigma.

Variables	Courtesy stigma		*p*
	Mean	SD	
Sex of children			0.721
Male	7.45	4.37	
Female	7.59	3.53	
Age of children (years)			0.655
<6	7.56	3.92	
≥6	7.37	4.53	
Duration of rehabilitation			0.507
<6 months	7.27	3.55	
6–12 months	7.20	3.47	
>1 years	7.71	4.61	
Comorbidities			0.153
Yes	8.57	4.57	
No	7.42	4.09	
Severity of symptoms			0.006
Mild	6.61	3.99	
Moderate	7.68	3.98	
Severe	8.38	4.51	
Single child			0.277
Yes	7.72	4.34	
No	7.29	3.92	
Picky eater			0.001
Yes	8.04	3.90	
No	6.67	4.33	

### Primary caregiver-related factors

The 428 primary caregivers who participated in this study ranged in age from 21 to 71 years, with a mean age of 34.36 ± 7.22 years. Almost two-thirds of the primary caregivers (64.02%) were female, and nearly three-fifths (58.88%) had below college degree education. Approximately one-third of the primary caregivers (34.58%) were fathers, 60.98% were mothers, and only 4.44% were grandparents of the children with ASD. More than half of the primary caregivers (54.44%) lived in urban areas and almost half (49.77%) were currently unemployed. More than two-fifths of primary caregivers (42.52%) had a monthly family income of ≤5,000 Yuan (Table 1). Univariate analyses indicated that there were significant differences in courtesy stigma scores among primary caregivers of children with ASD according to monthly family income, satisfaction with marital status, levels of challenge in caring for children, and economic burden (*p* < 0.05) (Table 3).

**Table 3:** Relationship between primary caregiver-related factors and courtesy stigma.

Variables	Courtesy stigma		*p*
	Mean	SD	
Sex of primary caregiver			0.576
Male	7.34	3.81	
Female	7.58	4.30	
Age of primary caregiver (years)			0.204
≤30	7.04	4.25	
31–45	7.60	4.14	
>45	8.36	3.43	
Education			0.090
Below college degree	7.21	4.35	
College or above degree	7.90	3.76	
Relationship to child with ASD			0.796
Father	7.32	3.88	
Mother	7.56	4.28	
Grandparent	7.84	4.03	
Area of residence			0.835
Urban	7.45	4.16	
Rural	7.54	4.10	
Employment status			0.303
Employed	7.70	3.86	
Unemployed	7.29	4.38	
Monthly family income (yuan)			0.032
≤5,000	7.48	4.35	
5,001–10,000	7.96	4.12	
>10,000	6.47	3.42	
Satisfaction with marital status			<0.001
Satisfaction	6.98	3.95	
Not too satisfaction	9.07	4.29	
Levels of challenge in caring for children			0.004
High	7.73	4.18	
Low	6.10	3.53	
Economic burden			<0.001
Severe	7.99	4.29	
Mild	6.52	3.60	

### Correlations of general self-efficacy and social support with courtesy stigma

Continuous data were tested for normality by analysis of kurtosis and skewness (50). The absolute kurtosis and skewness values of the studied variables were within 1.7 and 0.6, respectively, indicating that the data met the criteria for normal distribution. Among the respondents, mean ± SD general self-efficacy and social support scores were 26.36 ± 4.49 and 57.20 ± 13.71, respectively. Correlation analyses showed that both general self-efficacy and social support were negatively correlated with courtesy stigma of primary caregivers of children with ASD (*r* = −0.297 and *r* = −0.271, respectively, *p* < 0.001) (Table 4).

**Table 4:** Correlations of general self-efficacy and social support with courtesy stigma.

Variables	Mean ± SD	Correlation with courtesy stigma	
		Correlation coefficients	*p*
General self-efficacy	26.36 ± 4.49	−0.297	<0.001
Social support	57.20 ± 13.71	−0.271	<0.001

### Multiple linear regression analysis of factors associated with courtesy stigma

Multiple linear regression analysis indicated that the primary caregivers of children who were not picky eaters had lower courtesy stigma scores than those with picky eaters (*β* = −1.33, 95% CI: −2.08 – –0.58, *p* < 0.05). Primary caregivers who were not too satisfied with their current marital status had higher courtesy stigma scores than those who were satisfied (*β* = 1.21, 95% CI: 0.34–2.08, *p* < 0.05). Primary caregivers who reported a low level of challenge in caring for children with ASD had lower courtesy stigma scores than those who reported a high level of challenge (*β* = −1.16, 95% CI: −2.20 – –0.12, *p* < 0.05). In addition, primary caregivers with good general self-efficacy (*β* = −0.16, 95% CI: −0.25 – –0.06, *p* < 0.05) and social support (*β* = −0.04, 95% CI: −0.08 – –0.01, *p* < 0.05) had low courtesy stigma scores. Therefore, child picky eaters, satisfaction with marital status, challenges of caring for children, general self-efficacy, and social support may be useful for predicting courtesy stigma among primary caregivers of children with ASD (Table 5).

**Table 5:** Multiple linear regression model to determine the factors associated with courtesy stigma.

Variables	Estimate	95% CI		SE	*t*	*p*
		Lower	Upper			
Severity of symptoms (Ref: Mild)						
Moderate	0.50	−0.35	1.35	0.43	1.15	0.251
Severe	0.80	−0.32	1.92	0.57	1.40	0.162
Picky eater (Ref: yes)						
No	−1.33	−2.08	−0.58	0.38	−3.47	0.001
Monthly family income (yuan) (Ref: >10,000)						
≤5,000	−0.15	−1.24	0.94	0.55	−0.27	0.789
5,001–10,000	0.72	−0.35	1.78	0.54	1.32	0.187
Satisfaction with marital status (Ref: satisfaction)						
Not too satisfaction	1.21	0.34	2.08	0.44	2.74	0.006
Levels of challenge in caring for children (Ref: high)						
Low	−1.16	−2.20	−0.12	0.53	−2.19	0.029
Economic burden (Ref: severe)						
Mild	−0.41	−1.25	0.44	0.43	−0.94	0.346
General self-efficacy	−0.16	−0.25	−0.06	0.05	−3.30	0.001
Social support	−0.04	−0.08	−0.01	0.02	−2.79	0.005
Constant	15.78	12.25	19.30	1.79	8.80	<0.001

Ref: reference.

## Discussion

Stigma has been identified as a major problem experienced by the primary caregivers of children with ASD (42). This study addressed the gap in research on courtesy stigma among primary caregivers of children with ASD in eastern China, by determining the status of courtesy stigma among primary caregivers of children with ASD in Lianyungang, Jiangsu Province, Eastern China and identifying possible predictors. Our findings show that the mean ± SD score of the courtesy stigma scale was 7.49 ± 4.13, while the mean ± SD score for each item was 1.07 ± 0.59. Previous qualitative studies conducted in China have suggested that parents of children with ASD often experience courtesy stigma, due to their close relationship with the child (6). Therefore, there is an urgent need to develop effective interventions to help reduce courtesy stigma for primary caregivers of children with ASD. We found that the child-related factor, picky eating, was associated with courtesy stigma among primary caregivers of children with ASD. Primary caregiver-related factors, including satisfaction with marital status and level of challenge in caring for children were also associated with courtesy stigma, as were general self-efficacy and social support. In terms of child-related factors, our study showed that sex, age, duration of rehabilitation, comorbidities, severity of symptoms, and single child were not significantly associated with courtesy stigma. In terms of primary caregiver-related factors, sex, age, education level, relationship to children with ASD, area of residence, employment status, monthly family income, and economic burden were not significantly associated with courtesy stigma.

### Relationship of child-related factors with courtesy stigma

In this study, 69.63% of the children with ASD were boys, which is consistent with the sex distribution of ASD in epidemiological studies (51); however, we did not find any association between the sex of children with ASD and courtesy stigma of primary caregivers, consistent with a previous study conducted in Guangdong, China (37), but in contrast with a study conducted in eastern India, where parents of girls with ASD reported higher stigma scores (18). This may be due to differences in measurement instruments, sample size, and study subjects. This may also be due to variation in social and cultural background, such as political system and etiquette customs (41) and more extensive future studies in different regions are warranted. Children with ASD are often described as picky or selective eaters (52, 53). Most children with ASD show aversion to specific food colors, textures, odors, or other food characteristics (54). The results of this study suggest that primary caregivers of children with ASD who are picky eaters are more likely to experience courtesy stigma, possibly because they are more likely to perceive their children as unhealthy eaters or eating too few food types, and to have negative views of the eating behavior of their children (55). This may cause caregivers to feel that they will be perceived differently, thus increasing courtesy stigma. In addition, people may also perceive their children as “difficult” and/or “problematic,” and be critical of their ability to function as caregivers, making it possible for them to experience courtesy stigma in environments where support is expected (28). Children with ASD who are picky eaters can suffer from nutritional deficiencies, and persistent picky eaters may have developmental difficulties and gastrointestinal diseases (54, 56). Therefore, children with ASD who are picky eaters should be identified early, and strategies to improve picky eating provided for primary caregivers.

### Relationship of primary caregiver-related factors to courtesy stigma

Children with ASD can impact the healthy family structure and function, particularly the relationship between husband and wife (37). In this study, we found that marital status satisfaction was associated with courtesy stigma among primary caregivers of children with ASD, consistent with a previous report of an adverse association between maternal stigma and marital satisfaction (26). Studies in other populations have also shown that reduced marital satisfaction is associated with stronger self-reported stigma (57). Marital status satisfaction is one of the key factors in determining whether the family will be susceptible to disorganization when the whole family undertakes the task of child care (26). Higher marital status satisfaction may make primary caregivers more comfortable with difficulties associated with the presence of children with ASD, including stigma. Primary caregivers who were satisfied with their current marital status were more likely to have good communication, helpful interaction, emotional support, and accurate and useful information from their partners, which reduced their courtesy stigma to some extent; however, primary caregivers who were less satisfied with their current marital status were likely to experience more verbal aggression, intimidation, non-verbal anger, and disruptive interactions from their partners. Therefore, family-based interventions that promote effective communication, problem solving, and mutual support between couples are of some importance in reducing stigma. Raising a child with ASD requires a considerable amount of care, and many primary caregivers are forced to quit their jobs to care for their ASD child (42). This study demonstrated that primary caregivers who reported a higher level of challenge in caring for children with ASD were more likely to experience courtesy stigma. The high-level challenges experienced by primary caregivers caring for children with ASD may involve dealing with specific behaviors, such as screaming, aggression, and tantrums, which could lead to isolation and exclusion of primary caregivers, as well as difficulties in interacting with others in public places, thereby increasing courtesy stigma. A previous study demonstrated that parents experienced more stigma when their children exhibited higher level ASD-related behaviors (15). Therefore, clinical professionals should train primary caregivers of children with ASD in behavioral management and coping skills, to reduce the challenges of caring for children with ASD, thereby reducing courtesy stigma.

### Relationship of general self-efficacy and social support with courtesy stigma

In this study, we found that primary caregivers of children with ASD who had low general self-efficacy had higher courtesy stigma scores, similar to previous findings of a significant negative association between self-esteem and affiliate stigma (37); however, self-efficacy is a dynamic and modifiable characteristic. Therefore, measures should be implemented to improve the general self-efficacy of primary caregivers of children with ASD. It is necessary to help the main caregivers of children with ASD to establish self-understanding and evaluation, so that they realize that their own value, which could reduce their courtesy stigma. More support and counseling services for parents of children with ASD could reduce their stigma (6). Our findings show that social support was negatively correlated with courtesy stigma among primary caregivers of children with ASD. This finding is similar to previous literature showing that social support, particularly from family and friends, is associated with the mental health of mothers of children with ASD (40, 58). Social support can directly affect an individual’s well-being and can alleviate distress; therefore, it is important to implement specific interventions to increase social support, to reduce courtesy stigma among primary caregivers of children with ASD. In addition, it is necessary to educate the public about ASD and improve their understanding of ASD (6).

### Limitations

This study has important theoretical and practical implications, but also some limitations. First, the cross-sectional design of this study limits the inference of causality between variables, and future longitudinal studies are needed. Second, in this study, we recruited only primary caregivers of children with ASD who were undergoing rehabilitation in a rehabilitation facility, where primary caregivers would receive professional and peer support, which may have resulted in underestimation of courtesy stigma. Future studies are needed to expand the population to include primary caregivers of children with ASD outside rehabilitation facilities. Third, this study was restricted to Lianyungang, Jiangsu Province, Eastern China; hence, the results may only be representative of areas with similar backgrounds, and future studies in other regions are needed. Fourth, some variables in this study were measured by one item, and in the future multiple items or other assessment methods are needed. Finally, only quantitative analysis was conducted in this study, and we did not include qualitative analysis to provide a more comprehensive assessment of courtesy stigma. Future qualitative research is warranted.

## Conclusion

There is a high level of courtesy stigma among primary caregivers of children with ASD in eastern China. Children who are picky eaters, marital satisfaction of primary caregivers, the level of challenge in caring for children, general self-efficacy, and social support are factors associated with courtesy stigma. There is an urgent need to establish effective interventions to reduce the courtesy stigma among primary caregivers of children with ASD. Actions taken to address the associated factors discussed above may help to reduce courtesy stigma and its adverse consequences among primary caregivers of children with ASD.

## Data availability statement

The original contributions presented in the study are included in the article/supplementary material, further inquiries can be directed to the corresponding authors.

## Ethics statement

The studies involving humans were approved by the Ethics Committee of Lianyungang Maternal and Child Health Hospital. The studies were conducted in accordance with the local legislation and institutional requirements. The participants provided their written informed consent to participate in this study.

## Author contributions

XC, JT, DY, and LW designed and advanced the entire study. XC and JT collated, analyzed the data, and wrote the manuscript. All authors contributed to the article and approved the submitted version.

## References

[1] Lord C Cook EH Leventhal BL Amaral DG. Autism spectrum disorders. Neuron. (2000) 28:355–63. doi: 10.1016/s0896-6273(00)00115-x 11144346

[2] Salari N Rasoulpoor S Rasoulpoor S Shohaimi S Jafarpour S Abdoli N. The global prevalence of autism spectrum disorder: a comprehensive systematic review and meta-analysis. Ital J Pediatr. (2022) 48:112. doi: 10.1186/s13052-022-01310-w 35804408 PMC9270782

[3] Liu X Lin S Chen W Chan F Shen S Qiu X. Prevalence for autism spectrum disorders among children in China: a systematic review and meta-analysis. J Child Health Care. (2018) 26:402-406+429

[4] Liao X Lei X Li Y. Stigma among parents of children with autism: a literature review. Asian J Psychiatr. (2019) 45:88–94. doi: 10.1016/j.ajp.2019.09.007, PMID: 31542694

[5] Gill J Liamputtong P. Being the mother of a child with Asperger's syndrome: women's experiences of stigma. Health Care Women Int. (2011) 32:708–22. doi: 10.1080/07399332.2011.555830, PMID: 21767096

[6] Ng CSM Ng SSL. A qualitative study on the experience of stigma for Chinese parents of children with autism spectrum disorder. Sci Rep. (2022) 12:19550. doi: 10.1038/s41598-022-23978-0, PMID: 36379973 PMC9666461

[7] Crowell JA Keluskar J Gorecki A. Parenting behavior and the development of children with autism spectrum disorder. Compr Psychiatry. (2019) 90:21–9. doi: 10.1016/j.comppsych.2018.11.007 30658339

[8] Werner S Shulman C. Does type of disability make a difference in affiliate stigma among family caregivers of individuals with autism, intellectual disability or physical disability? J Intellect Disabil Res. (2015) 59:272–83. doi: 10.1111/jir.12136 24761747

[9] Zhou T Wang Y Yi C. Affiliate stigma and depression in caregivers of children with autism Spectrum disorders in China: effects of self-esteem, shame and family functioning. Psychiatry Res. (2018) 264:260–5. doi: 10.1016/j.psychres.2018.03.071, PMID: 29655969

[10] Grover S Avasthi A Singh A Dan A Neogi R Kaur D. Stigma experienced by caregivers of patients with severe mental disorders: a nationwide multicentric study. Int J Soc Psychiatry. (2017) 63:407–17. doi: 10.1177/0020764017709484, PMID: 28537123

[11] Zuckerman KE Sinche B Mejia A Cobian M Becker T Nicolaidis C. Latino parents' perspectives on barriers to autism diagnosis. Acad Pediatr. (2014) 14:301–8. doi: 10.1016/j.acap.2013.12.004.C, PMID: 24767783 PMC4006363

[12] Zhou T Yi C. Parenting styles and parents' perspectives on how their own emotions affect the functioning of children with autism spectrum disorders. Fam Process. (2014) 53:67–79. doi: 10.1111/famp.12058, PMID: 24400727

[13] Chan KKS Leung DCK Fung WTW. Longitudinal impact of parents' discrimination experiences on children's internalizing and externalizing symptoms: a 2-year study of families of autistic children. Autism. (2023) 27:296–308. doi: 10.1177/13623613221093110, PMID: 35585707

[14] Chan KKS Leung DCK. Linking child autism to parental depression and anxiety: the mediating roles of enacted and felt stigma. J Autism Dev Disord. (2021) 51:527–37. doi: 10.1007/s10803-020-04557-6, PMID: 32519191

[15] Kinnear SH Link BG Ballan MS Fischbach RL. Understanding the experience of stigma for parents of children with autism Spectrum disorder and the role stigma plays in Families' lives. J Autism Dev Disord. (2016) 46:942–53. doi: 10.1007/s10803-015-2637-9, PMID: 26659549

[16] Farrugia D. Exploring stigma: medical knowledge and the stigmatisation of parents of children diagnosed with autism spectrum disorder. Sociol Health Illn. (2009) 31:1011–27. doi: 10.1111/j.1467-9566.2009.01174.x, PMID: 19659737

[17] Ji B Jiang X Luo Y. Autistic children's age difference in affiliate stigma and resilience of their parents in China: a cross-sectional study. Arch Psychiatr Nurs. (2022) 39:7–12. doi: 10.1016/j.apnu.2022.01.006, PMID: 35688547

[18] Patra S Kumar PB. Affiliate stigma among parents of children with autism in eastern India. Asian J Psychiatr. (2019) 44:45–7. doi: 10.1016/j.ajp.2019.07.018, PMID: 31315059

[19] Cantwell J Muldoon O Gallagher S. The influence of self-esteem and social support on the relationship between stigma and depressive symptomology in parents caring for children with intellectual disabilities. J Intellect Disabil Res. (2015) 59:948–57. doi: 10.1111/jir.12205, PMID: 26031805

[20] Wong CCY Mak WWS Liao KY-H. Self-compassion: a potential buffer against affiliate stigma experienced by parents of children with autism Spectrum disorders. Mindfulness. (2016) 7:1385–95. doi: 10.1007/s12671-016-0580-2

[21] Chan KKS Lam CB. Trait mindfulness attenuates the adverse psychological impact of stigma on parents of children with autism Spectrum disorder. Mindfulness. (2017) 8:984–94. doi: 10.1007/s12671-016-0675-9

[22] Pohl AL Crockford SK Blakemore M Allison C Baron-Cohen S. A comparative study of autistic and non-autistic women's experience of motherhood. Mol Autism. (2020) 11:3. doi: 10.1186/s13229-019-0304-2, PMID: 31911826 PMC6945630

[23] Pyszkowska A Rożnawski K Farny Z. Self-stigma and cognitive fusion in parents of children with autism spectrum disorder. The Moderating role of self-compassion. PeerJ. (2021) 9:e12591. doi: 10.7717/peerj.12591, PMID: 35003921 PMC8684717

[24] Yip CCH Chan KKS. Longitudinal impact of public stigma and courtesy stigma on parents of children with autism spectrum disorder: the moderating role of trait mindfulness. Res Dev Disabil. (2022) 127:104243. doi: 10.1016/j.ridd.2022.104243, PMID: 35661545

[25] Zuckerman KE Lindly OJ Reyes NM Chavez AE Cobian M Macias K. Parent perceptions of community autism Spectrum disorder stigma: measure validation and associations in a multi-site sample. J Autism Dev Disord. (2018) 48:3199–209. doi: 10.1007/s10803-018-3586-x, PMID: 29700707 PMC7466820

[26] Kwok SY Leung CL Wong DF. Marital satisfaction of Chinese mothers of children with autism and intellectual disabilities in Hong Kong. J Intellect Disabil Res. (2014) 58:1156–71. doi: 10.1111/jir.12116, PMID: 24450394

[27] Siman-Tov A Kaniel S. Stress and personal resource as predictors of the adjustment of parents to autistic children: a multivariate model. J Autism Dev Disord. (2011) 41:879–90. doi: 10.1007/s10803-010-1112-x, PMID: 20872059

[28] Cunliffe L Coulthard H Williamson IR. The lived experience of parenting a child with sensory sensitivity and picky eating. Matern Child Nutr. (2022) 18:e13330. doi: 10.1111/mcn.13330 35195333 PMC9218328

[29] Waluyo A Mansyur M Earnshaw VA Steffen A Herawati T Maria R. Exploring HIV stigma among future healthcare providers in Indonesia. AIDS Care. (2022) 34:29–38. doi: 10.1080/09540121.2021.1897777, PMID: 33715515

[30] He D He L Yuan Y Huang L Xiao Q Ye X. Stigma and its correlates among patients with Crohn's disease: a cross-sectional study in China. Int J Nurs Sci. (2023) 10:318–24. doi: 10.1016/j.ijnss.2023.06.012, PMID: 37545781 PMC10401350

[31] Zhang F Lv Y Wang Y Cheng X Yan Y Zhang Y. The social stigma of infertile women in Zhejiang Province, China: a questionnaire-based study. BMC Womens Health. (2021) 21:97. doi: 10.1186/s12905-021-01246-z, PMID: 33663480 PMC7934237

[32] Liu X Yin M Li Z Wang D. Psychosocial correlates of internalized stigma among Chinese individuals with severe mental illness. J Psychosoc Nurs Ment Health Serv. (2023) 1–8. doi: 10.3928/02793695-20230726-05, PMID: 37527516

[33] Qin Y Dai M Chen L Zhang T Zhou N Chen X. The relationship between ecological executive function and stigma among patients with epilepsy: the mediating effect of social support. Epilepsy Res. (2022) 182:106919. doi: 10.1016/j.eplepsyres.2022.106919, PMID: 35427990

[34] Huang XQ Zhang H Chen S. Neuropsychiatric symptoms, parenting stress and social support in Chinese mothers of children with autism Spectrum disorder. Curr Med Sci. (2019) 39:291–7. doi: 10.1007/s11596-019-2033-3, PMID: 31016524

[35] Zablotsky B Bradshaw CP Stuart EA. The association between mental health, stress, and coping supports in mothers of children with autism spectrum disorders. J Autism Dev Disord. (2013) 43:1380–93. doi: 10.1007/s10803-012-1693-7, PMID: 23100053

[36] Zhang J Schwarzer R. Measuring optimistic self-beliefs: a Chinese adaptation of the general self-efficacy scale. Psychol Int J Psychol Orient. (1995) 38:174–81.

[37] Lyu QY Yu XX Wang JL Wang XY Ke QQ Liu D. Self-esteem and family functioning mediates the association of symptom severity and parental affiliate stigma among families with children with ASD. J Pediatr Nurs. (2022) 66:e122–9. doi: 10.1016/j.pedn.2022.04.019, PMID: 35537979

[38] Drogomyretska K Fox R Colbert D. Brief report: stress and perceived social support in parents of children with ASD. J Autism Dev Disord. (2020) 50:4176–82. doi: 10.1007/s10803-020-04455-x, PMID: 32200467

[39] Wang YZ Meng XD Zhang TM Weng X Li M Luo W. Affiliate stigma and caregiving burden among family caregivers of persons with schizophrenia in rural China. Int J Soc Psychiatry. (2023) 69:1024–32. doi: 10.1177/00207640231152206, PMID: 36708508

[40] Kulasinghe K Whittingham K Mitchell AE. Mental health, broad autism phenotype and psychological inflexibility in mothers of young children with autism spectrum disorder in Australia: a cross-sectional survey. Autism. (2021) 25:1187–202. doi: 10.1177/1362361320984625, PMID: 33504195

[41] Mak WWS Kwok YTY. Internalization of stigma for parents of children with autism spectrum disorder in Hong Kong. Soc Sci Med. (1982). 2010) 70:2045–51. doi: 10.1016/j.socscimed.2010.02.023, PMID: 20362379

[42] Alshaigi K Albraheem R Alsaleem K Zakaria M Jobeir A Aldhalaan H. Stigmatization among parents of autism spectrum disorder children in Riyadh, Saudi Arabia. Int. J Pediat Adol Med. (2020) 7:140–6. doi: 10.1016/j.ijpam.2019.06.003, PMID: 33094144 PMC7568054

[43] Hwang K-K. Moral face and social face: contingent self-esteem in confucian society. Int J Psychol. (2006) 41:276–81. doi: 10.1080/00207590544000040

[44] Papadopoulos C Foster J Caldwell K. 'Individualism-collectivism' as an explanatory device for mental illness stigma. Community Ment Health J. (2013) 49:270–80. doi: 10.1007/s10597-012-9534-x, PMID: 22837106

[45] Struening EL Perlick DA Link BG Hellman F Herman D Sirey JA. Stigma as a barrier to recovery: the extent to which caregivers believe most people devalue consumers and their families. Psychiatr Serv. (2001) 52:1633–8. doi: 10.1176/appi.ps.52.12.1633, PMID: 11726755

[46] Hammersland MH Aarsand AK Sandberg S Andersen J. Self-efficacy and self-management strategies in acute intermittent porphyria. BMC Health Serv Res. (2019) 19:444. doi: 10.1186/s12913-019-4285-9, PMID: 31269991 PMC6607542

[47] Arima M Takamiya Y Furuta A Siriratsivawong K Tsuchiya S Izumi M. Factors associated with the mental health status of medical students during the COVID-19 pandemic: a cross-sectional study in Japan. BMJ Open. (2020) 10:e043728. doi: 10.1136/bmjopen-2020-043728, PMID: 33303472 PMC7733210

[48] Dahlem NW Zimet GD Walker RR. The multidimensional scale of perceived social support: a confirmation study. J Clin Psychol. (1991) 47:756–61. doi: 10.1002/1097-4679(199111)47:6<756::aid-jclp2270470605>3.0.co;2-l 1757578

[49] Singh P Ghosh S Nandi S. Subjective burden and depression in mothers of children with autism Spectrum disorder in India: moderating effect of social support. J Autism Dev Disord. (2017) 47:3097–111. doi: 10.1007/s10803-017-3233-y, PMID: 28695439

[50] Qiu L Yang Q Tong Y Lu Z Gong Y Yin X. The mediating effects of stigma on depressive symptoms in patients with tuberculosis: a structural equation modeling approach. Front Psych. (2018) 9:618. doi: 10.3389/fpsyt.2018.00618, PMID: 30534088 PMC6275230

[51] Christensen DL Baio J Van Naarden Braun K Bilder D Charles J Constantino JN. Prevalence and characteristics of autism Spectrum disorder among children aged 8 years--autism and developmental disabilities monitoring network, 11 sites, United States, 2012. MMWR Surveill Summ. (2016) 65:1–23. doi: 10.15585/mmwr.ss6503a1 PMC7909709 27031587

[52] Cermak SA Curtin C Bandini LG. Food selectivity and sensory sensitivity in children with autism spectrum disorders. J Am Diet Assoc. (2010) 110:238–46. doi: 10.1016/j.jada.2009.10.032, PMID: 20102851 PMC3601920

[53] Berding K Donovan SM. Diet can impact microbiota composition in children with autism Spectrum disorder. Front Neurosci. (2018) 12:515. doi: 10.3389/fnins.2018.00515, PMID: 30108477 PMC6079226

[54] Ristori MV Quagliariello A Reddel S Ianiro G Vicari S Gasbarrini A. Autism, gastrointestinal symptoms and modulation of gut microbiota by nutritional interventions. Nutrients. (2019) 11:2812. doi: 10.3390/nu11112812, PMID: 31752095 PMC6893818

[55] Lockner DW Crowe TK Skipper BJ. Dietary intake and parents' perception of mealtime behaviors in preschool-age children with autism spectrum disorder and in typically developing children. J Am Diet Assoc. (2008) 108:1360–3. doi: 10.1016/j.jada.2008.05.003, PMID: 18656577

[56] Taylor CM Emmett PM. Picky eating in children: causes and consequences. Proc Nutr Soc. (2019) 78:161–9. doi: 10.1017/s0029665118002586, PMID: 30392488 PMC6398579

[57] Dirkse D Lamont L Li Y Simonič A Bebb G Giese-Davis J. Shame, guilt, and communication in lung cancer patients and their partners. Curr Oncol. (2014) 21:e718–22. doi: 10.3747/co.21.2034, PMID: 25302043 PMC4189577

[58] Benson PR Karlof KL. Anger, stress proliferation, and depressed mood among parents of children with ASD: a longitudinal replication. J Autism Dev Disord. (2009) 39:350–62. doi: 10.1007/s10803-008-0632-0, PMID: 18709548

